# Detecting event-related changes in organizational networks using optimized neural network models

**DOI:** 10.1371/journal.pone.0188733

**Published:** 2017-11-30

**Authors:** Ze Li, Duoyong Sun, Renqi Zhu, Zihan Lin

**Affiliations:** College of Information System and Management, National University of Defense Technology, Changsha, Hunan, China; Southwest University, CHINA

## Abstract

Organizational external behavior changes are caused by the internal structure and interactions. External behaviors are also known as the behavioral events of an organization. Detecting event-related changes in organizational networks could efficiently be used to monitor the dynamics of organizational behaviors. Although many different methods have been used to detect changes in organizational networks, these methods usually ignore the correlation between the internal structure and external events. Event-related change detection considers the correlation and could be used for event recognition based on social network modeling and supervised classification. Detecting event-related changes could be effectively useful in providing early warnings and faster responses to both positive and negative organizational activities. In this study, event-related change in an organizational network was defined, and artificial neural network models were used to quantitatively determine whether and when a change occurred. To achieve a higher accuracy, Back Propagation Neural Networks (BPNNs) were optimized using Genetic Algorithms (GAs) and Particle Swarm Optimization (PSO). We showed the feasibility of the proposed method by comparing its performance with that of other methods using two cases. The results suggested that the proposed method could identify organizational events based on a correlation between the organizational networks and events. The results also suggested that the proposed method not only has a higher precision but also has a better robustness than the previously used techniques.

## 1. Introduction

Researchers aim to analyze social organizations using quantitative models. For instance, Social Network Analysis (SNA) is a general framework that has been widely and extensively applied in many fields. SNA is an interdisciplinary research approach that aims to interpret the structure of the relationships among social entities and the effect of this structure on other social phenomena. Current studies indicate that SNA is a useful tool for understanding the structure and pattern of a social organization [1]. However, realistic organizations evolve dynamically and change constantly [2,3]. For example, as new information arrives, nodes and links can appear or disappear, and the strength of the links can change. Therefore, SNA has shortcomings because it focuses primarily on a static analysis. To solve this problem, a new sub-field, dynamic network analysis has been proposed in which the evolution process is represented by temporal sequence networks. Dynamic network analysis focuses on collecting, understanding and predicting dynamic relationships and the effects of these dynamics on the behavior of individuals and groups [2]. Currently, many corresponding technologies and research methods for investigating dynamic networks are available [2,4–7]. Dynamic social network change detection, which is also known as anomaly detection or change-point detection, is the study task that has received the most attention.

Change detection is important because in reality, changes in networks and their behaviors translate to significant (and often critical) events for the organization. Many approaches have been effectively employed by various researchers and research groups to achieve this goal [4–6]. Studies have shown that a correlation exists between changes in an organizational network and the emergence of organizational behaviors. Specifically, an anomalous change in the network internal structure usually leads to external events, and when an external event occurs, the network internal structure becomes anomalous compared with its normal patterns. For example, changes in the patterns of interactions among members in an e-mail network may be associated with an important event of a public company [5,8]. An anomalous terrorist network structure could be indicative of the potential planning or performing of attacks by an organization [4,9]. However, previous studies have ignored this correlation and focused on detecting the anomalous organizational networks rather than the anomalous behaviors. Although the correlation has been shown, these studies fail to employ the correlation as an input factor. Previous studies have defined “change” as a network with abnormal patterns or structure. Thus, a change in the organizational network indicates an underlying change within the organization and the possibility of significant events or behaviors. There are many types of abnormal patterns or behaviors in an organizational network. However, in this study, rather than detecting all changes, we focused on detecting those changes that are highly related to external events. Detecting event-related changes in social networks over time can help identify the external events of an organization in a timely manner and provide a better understanding of the organizational behavior pattern. Furthermore, change patterns can provide an early warning of social anomalies caused by natural or man-made disasters.

To detect event-related changes in organizational networks, we must provide a definition for “event-related change”, devise an approach that will address the correlation, and design a model that efficiently detects the changes. Omitting any of these elements substantially reduces the chance that the events will be detected. In this study, we combined the SNA, anomaly detection, and supervised classification approaches to construct a novel research framework. Specifically, we first provided a definition for event-related change. Then, we applied optimized artificial neural networks to detect event-related changes in organizational social networks by considering the correlation between the network internal structure and external events. In this paper, we designed a method with clear scientific implications. First, the use of an optimized neural network as a supervised method that considers the correlation between organizational internal networks and external events is an innovation in dynamic social network change detection. Second, social network-based change detection could be a potential application domain in artificial neural networks.

The remainder of this paper is organized as follows. First, an overview of related studies, as well as the research gaps and our research questions are presented in Section 2. A definition and problem description of event-related change detection, as well as the research framework are introduced in Section 3. Section 4 discusses our detection methodology of optimized neural network models. Section 5 introduces the experimental design. Section 6 presents our results and two cases. Section 7 draws conclusions and explores future directions.

## 2. Related studies

Studies related to social network-based change detection can be categorized into two groups, i.e., anomaly detection and classification methods. The related techniques are based the assumption that the changes in an organization are caused by predictable internal factors. Social network anomaly detection has been studied in a static network setting [10] using a general evolving dataset with multiple snapshots. The main goal of static detection is identify a small percentage of nodes or communities in the whole network [11]. In contrast, dynamic detection aims to identify networks in a temporal sequence whose movement patterns are different from those in the general population [12]. Priebe et al. [8] introduce the theory of Scan Statistics using graphs and apply their ideas to the problem of anomaly detection using a time series of Enron e-mail graphs. McCulloh and Carley [4] combine Statistical Process Control (SPC) and SNA and find that the Cumulative Sum (CUSUM) is a reasonable method for detecting changes in longitudinal social networks. Sun et al. [13] present a method called GraphScope, which is based on minimum description lengths, to detect when and how communities change in evolving networks. Gupta et al. [11] propose an effective two-step procedure to detect community trend outliers. Peel and Clauset [5] formalize the network change-point detection problem within a probabilistic framework and introduce the Generalized Hierarchical Random Graph (GHRG) model with the Generalized Likelihood Ratio (GLR) test to quantitatively determine whether, when, and precisely how a change point has occurred. Silva and Willett [14] perform a robust statistical analysis Expectation-Maximization algorithm to detect anomalous meetings in social networks. Heard et al. [15] present a simple statistical framework using Bayesian methods to identify anomalous network structures by monitoring dynamic social networks within a statistical distribution.

Other studies focus on classification methods using extracted features to understand the dynamics of a network. For a continuous organization, the behavior of the network is influenced by both the interactional environment and the organizational mechanism. Specifically, Vigliotti and Hankin [16] classify the behavior of individuals in temporal datasets as *normal* and *abnormal*. These authors first label certain individuals as abnormal and then present an approach to infer the subset of individuals that might share the same qualitative connotation. Raghavan et al. [17] quantitatively classify the group dynamics as *active* and *inactive* and use a Hidden Markov Model (HMM) to track and predict the state of groups. Wu et al. [18] introduce a framework for tracking the event-based evolution in social networks. Asur et al. [7] present an event-based characterization of critical behavioral patterns in temporally varying interaction graphs. Other studies focus on applying machine learning techniques, such as neural networks, to classify organizational behavior. Subrahmanian et al. [19] introduce Temporal Probabilistic (TP) rules and machine leaning techniques to detect and predict major terrorist attacks by the Pakistani-backed terrorist group Lashkar-e-Taiba. Furthermore, these authors develop a Stochastic Temporal Analysis of Terrorist Events (STATE) system based on the IP rule-mining engine for the early detection of terrorist attacks by Indian Mujahideen [20]. Xue et al. [21] propose the prediction algorithm PBCS, which is based on the context subspace. The proposed algorithm first extracts the context subspace according to the association between the context attributes and the behavior attributes; then, the algorithm predicts the organizational behavior based on the extracted context subspace. Sliva et al. [22] develop an architecture and algorithms called CAPE to detect changes in group behavior by including environmental conditions as input variables.

The literature on change detection could be viewed as researchers’ efforts to identify changes in social networks and explore their implications. We have become inspired by these studies. First, it has been established that SNA is a powerful tool capable of providing a predictive and explanatory value to the field of organizational behavior. Social structure-based networks that focus on individuals and the constituents of organizations are valuable for analyzing organizational behavior produced by individual interactions. Second, related studies have provided important insights into the critical correlation between events and the social network and revealed meaningful properties in actual dynamic behaviors. Thus, the relationships and interactions among the nodes at the micro level lead to the continuous development of the group behavior at the macro level. Third, machine leaning and classification approaches can be used to detect organizational behavior once the useful features are extracted.

However, previous studies have also left gaps. First, previous studies of anomaly detection using evolving datasets have focused on detecting anomalies or outliers in the internal network of an organization rather than on external events. As mentioned previously, few studies consider the correlation between the social networks and events. In fact, most studies use unsupervised anomaly detection methods without using external events as inputs. Second, previously used techniques rely solely on the specific structure and degree of distribution of the network. Therefore, these techniques less universality when used to analyze different datasets. In addition, these methods are weak because most of these methods process a single input (feature or measure), which does not adequately represent the whole network because some crucial information is discarded, and other measures are not considered. Third, no study combining the SNA and classification methods to provide a better method for the detection of event-related change has been reported in the literature. The supervised classification method using the correlation as an input factor remains a novel and challenging problem.

Based on these gaps in the literature, we form the crux of our study with the following questions:

*Q1*: *How can an event-related change in an organizational network be defined and detected*?
*Q2*: *Which technique is the most valuable for detecting event-related change in an organizational network*?
*Q3*: *How effective is our detection technique*?

To answer these questions, in the following sections, we propose an event-related change detection approach using supervised classification models. We hypothesize that it is possible to detect correlation-based event-related change by monitoring an organizational network. We optimize Back Propagation Neural Networks (BPNNs) using hybrid heuristic methods as supervised classification models to detect event-related changes. We demonstrate the effectiveness of our methods by comparing the results of two cases using different methods. We hypothesize that the proposed method not only has a higher precision but also has a better robustness than the previously used techniques.

## 3. Problem description and research framework

### 3.1 Definition and problem description

A social network is employed to model the structure of an organization based on the relationships among its members [1]. As social systems, organizations change dynamically because of internal interactions and the external environment. Thus, organizational social networks are constantly changing. For example, during the growing phase of an organization, the nodes, links, and the strength of those links can increase, whereas during the decay stage, the nodes and links can decrease, and the strength of the links can decrease. Several types of changes in organizational networks are reported in the dynamics literature. McCulloh and Carley [4] select the following four types of dynamic network behaviors to investigate network change: network stability, endogenous change, exogenous change, and initiated change.

However, as previously mentioned, we are not concerned with all these changes but rather only with those that are highly related to external events. Inspired by previous studies, we investigate event-related change in this study. Here, event-related changes are defined as changes in an organizational network that occur when an organization performs an external event or suffers from an exogenous incident. Here, these changes are contingent upon an organization having a significant event. The external event and exogenous incident are considered an event of the organization with no difference on the organization. When such an event affects the organization, the corresponding social network changes synchronously. This phenomenon can be interpreted as a strong correlation between the network internal structure and the external event. Identifying organizational events can be challenging, but identifying organizational network changes is relatively straightforward. By detecting the event-related changes, we can most likely identify the events of the organization. The definition of “change” provided in previous studies largely focuses on change in a network’s structure and topology. Here, we provide a definition for event-related change using different dimensions. We overlook the structure and topology and focus on whether there is an event that is relevant to the organization. This approach is relevant for the core principle of event-related change detection.

The primary goal of the event-related change detection problem is to recognize the pattern of the network as the network changes. Because we use classification methods to detect such changes, a network is an event-related change if its feature set matches the anomalous pattern. The change detection approach aims to define a region representing the normal pattern and identify any observations in the data that do not belong to this normal region [12]. The network measure can express the pattern to a certain extent. Each class of networks presents specific topological features that characterize its connectivity and highly influence the dynamics of the processes executed on the network [23]. Thus, our focus is on capturing the features of measures. Network measures perform differently with different datasets; however, the differences remain obvious between measures of normal and anomalous networks. The measures are potentially useful for characterizing the patterns of an organization, and the differences can eventually be used to distinguish event-related changes. Using change-based detection network measures, social scientists can also identify potential internal causes for an organizational event.

### 3.2 Research framework

**Fig 1:**
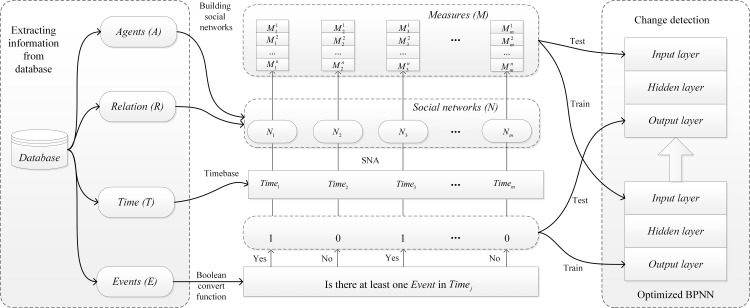
Research framework for detecting event-related changes in organizational networks.

In this study, we consider event-related change detection a classification problem and introduce supervised methods, i.e., optimized BPNN models, to identify event-related changes in organizational networks. The research framework is shown in Fig 1.

First, we extract *Agent*, *Relation*, *Time*, and *Event* from the database. *Agents* denote the individuals within an organization, while *Relation* denotes the relationship between each pair of *Agents*. *Events* are the known significant events of the organization. We extract the *Time* sequence from a continuous perspective with equal time intervals. For different datasets, the interval could be one hour, one day, one month, or even one year. Usually, the smaller the interval, the higher the precision.

Second, we build and analyze the social networks using SNA. At *Time*_*j*_ (*j* = 1,2,…*m*), given the information of *Agent* and *Relation*, we can build social network *N*_*j*_ of the organization. Then, we build a sequence of *m* networks, [*N*_1_,*N*_2_,…,*N*_*m*_]. Using SNA, we can obtain the multidimensional measure vector $\vec{M}$ ($\vec{M}={\left[M^{1},M^{2},\ldots ,M^{n}\right]}^{T}$) of each network.

Third, the time sequence is labeled with a 1/0 set based on whether the organization has an event. If the organization has a verified event, we consider the behavior of the organization anomalous; then, the corresponding network is classified into the class of networks with event-related change. Otherwise, the network is classified as a normal network. We discuss the classification approach in detail in Section 3.3.

### 3.3 Design of the classification approach

**Fig 2:**
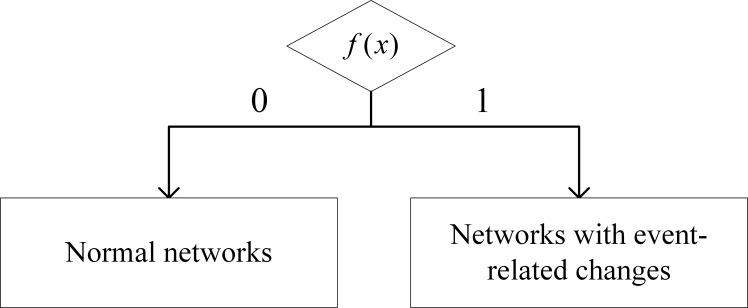
Classification approach for change detection.

The main purpose of our study is to classify the time sequence of organizational dynamic networks into the binary label, i.e., 0/1. As shown in Fig 1, we use the Boolean value convert function to assign a binary label to the time sequence:

$$Time_{j}=\{\begin{array}{l} 1,\;\mathrm{if}\;\mathrm{there}\;\mathrm{is}\;\mathrm{at}\;\mathrm{least}\;\mathrm{one}\;Event\;\mathrm{in}\;Time_{j},\\ 0,\;\mathrm{otherwise}. \end{array}$$ (1)

where *Time*_*j*_ ∈ {0, 1} is the classification label. Using this function, the time sequence *T* is converted into a set *T*′ with a dichotomous value (0/1). Then, the corresponding social network sequence *N* can also be converted into a labeled set *N*′. Suppose there are *m* networks included in the time sequence and $\left\{({\vec{M}}_{j},N_{j})\;|j=1,2,\ldots ,m\right\}$, where ${\vec{M}}_{j}$ denotes the extracted measures associated with the *j*-th network, and *N*_*j*_ ∈ {0, 1} is the classification label, such that *N*_*j*_ = 1 if and only if the network *N*_*j*_ has an event-related change at *Time*_*j*_. Our objective is to develop a detection model

$$f:\vec{M}\mapsto N^{\prime }=f(x)$$ (2)

that can be used to identify event-related networks based on the network sequence. The detecting function *f*(*x*) is illustrated in Fig 2.

We use the function *f*(*x*) to filter the networks with event-related changes based on the network sequence (i.e., *f*(*x*) = 1). Specifically, to distinguish networks with event-related changes from normal networks with a high accuracy and a low rate of false-positives, we use the optimized BPNN method as *f*(*x*). We first divide the population into the training and test samples. During the training phase, both the features extracted from the network ($\vec{M}$) and those extracted from the external event (labeled set *N*′) are imported as inputs into the classifiers (optimized BPNNs). After the training, the classifier can determine which types of features can lead to an anomalous or normal pattern. In contrast, during the test phase, we only input $\vec{M}$ of the network into the classifier. Then, the classifier can detect whether a network has an event-related change by matching its feature set to the patterns. Using this classification approach, event-related changes can be detected, and Question 1 in Section 2 can be answered. The following section provides details regarding how we optimized the BPNN to meet the needs of this study.

## 4. Optimized neural network models

**Fig 3:**
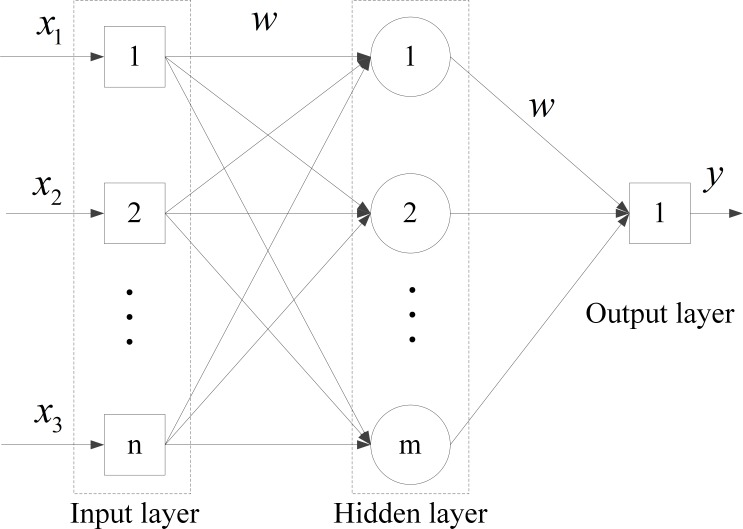
Typical topology of a BPNN.

The core component of our framework is the filtering of social networks with event-related change using BPNNs. Many general-purpose classification algorithms can be used to implement the filtering function *f*(*x*) in Eq (2). In this paper, BPNNs are selected as *f*(*x*) due to their superior detection accuracy, computing efficiency, and modeling flexibility. Fig 3 shows a typical topology of a BPNN model [24]. As a feed-forward network, a BPNN has two phases of non-linear transformations. The first phase (forward propagation) yields input information. Based on the input layer, the network calculates the actual output value of each unit layer by layer through hidden layer(s) [25]. If the expected output is not obtained in the output layer, the second phase (back propagation) is initiated. During the second phase, the differences between the actual outputs and expected outputs are calculated recursively, layer by layer. Then, the internal connection weights are adjusted based on the difference. The overall aim is to minimize the global difference.

However, a BPNN has limitations when it is applied to complex nonlinear optimization problems. Because numerous network measures are employed as input layer variables, a BPNN used in our event-related change detection problem will have two significant drawbacks, i.e., slowness in convergence and the inability to escape the local optima [24]. According to the features of the study content in this paper, BPNN models are optimized using heuristic methods, Genetic Algorithms (GAs) and Particle Swarm Optimization (PSO) to overcome the local convergence problem in nonlinear optimization problems.

### 4.1 Optimization using GA (GABP)

GAs simulate the mechanisms of population genetics and natural rules of survival in pursuit of the ideas of adaptation [26]. GAs possess good global optimum performance and global search ability. The GA-based search, occasionally with modifications to the simple GA formulation, has been shown to perform effectively in several applications. The model obtained indicated the high-quality performance of the neural network and its ability to identify the critical composition factors [27]. A general flowchart of GAs primarily contains the following three steps: first, initialize the candidate population; second, compute the fitness of all individuals and perform the selection; and third, apply genetic operators, including crossover and mutation, to generate offspring individuals. Generally, after several generations, the candidate solutions will converge toward the optimum.

**Fig 4:**
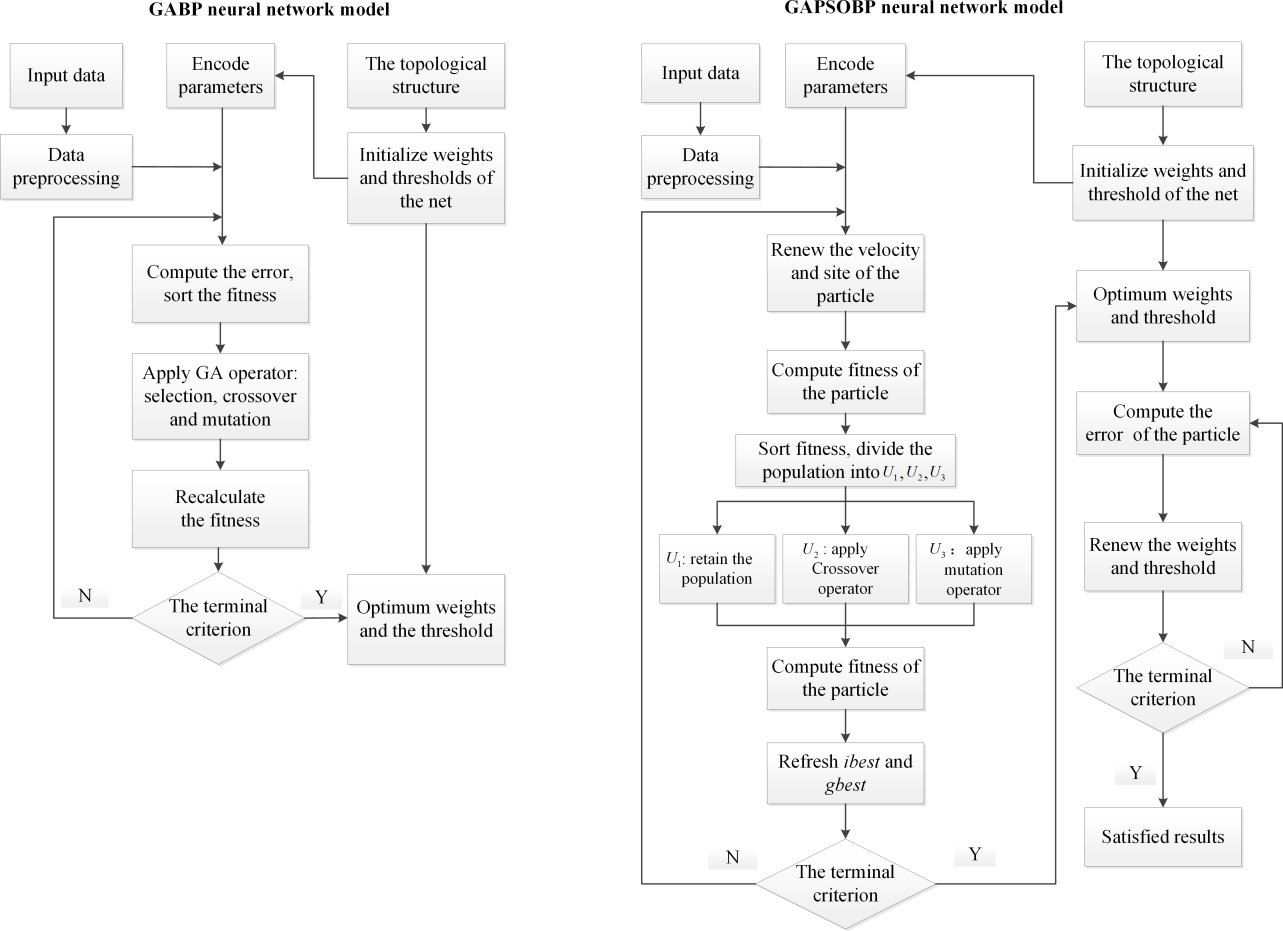
Flowchart of the proposed models.

GAs introduce the principle of survival of the fittest in nature for optimizing the parameters of coding populations. By combining GAs and a BPNN, the weights and thresholds of the neural network are optimized by the GAs; thus, GABP improves the convergence speed of the neural network and decreases the possibility of the algorithm converging into a local optimum [28]. The GA-optimized BPNN contains three parts. First, the structure of the BP is determined by the number of input parameters, which also stipulates the algorithm length in the BPNN part. Then, a GA optimizes the value of the weights and thresholds of the BP. All threshold and weight values in a neural network are incorporated into each individual in the species. The adaptive value of a function is calculated using the individual fitness function. The best individual is identified using the genetic operations of selection, crossing and mutation. Using a GA, the weights and thresholds are optimized with BPNN for prediction. Finally, after the training of the BPNN, a predictive output function should be obtained [29]. The procedure of the GABP method is illustrated in Fig 4.

### 4.2 Optimization using GA and PSO (GAPSOBP)

PSO was proposed by Eberhart and Kennedy [30] in 1995. PSO is also population based. However, unlike GAs, PSO algorithms typically have no crossover or mutation operators; instead, the particles (candidate solutions) update themselves according to both the local and global best particles under some rules. In a PSO system, each candidate solution is called a particle. During the search, each particle will update its site according to its historical best site and the global best site. Currently, many scholars employ PSO algorithms to optimize BPNNs, and good results are obtained. Specifically, the updating principle is expressed as follows [31].

$$v_{i\cdot d}=w\cdot v_{i\cdot d}+c_{1}\cdot rand(pbest_{i\cdot d}-x_{i\cdot d})+c_{2}\cdot rand\cdot (gbest+x_{i\cdot d})$$ (3)

$$x_{i\cdot d}=x_{i\cdot d}+v_{i\cdot d}$$ (4)

The current position of particle *i* is denoted by *x*_*i*⋅*d*_ = [*x*_*i*⋅1_,*x*_*i*⋅2_,…,*x*_*i*⋅*D*_], *i* = 1,2,…,*D*, where *M* is the size of the swarm, and *D* is the dimension of the solution space. The current velocity of particle *i* is denoted as *v*_*i*⋅*d*_, where *d* = 1,2,…,*D*; *rand* denotes a random number in [0, 1]; *c*_1_ and *c*_2_ represent two acceleration coefficients; *w* represents the inertia weight; *pbest*_*i*⋅*d*_ represents the best position found by a particle thus far; and *gbest* represents the global best position found in the neighborhood of the particle.

The first part of Eq (3) indicates the previous velocity, which supplies the particles with the momentum necessary to pass through the search space. The second part is called the “cognitive” component, which represents the personal thinking of a particle. The cognitive component brings the particle to the best position found thus far as the desired input to make the particle move toward its own best position. The third part is named the “social” component, which represents the collaborative behavior of the particles to find the global optimal solution. The social component motivates the particles to the best position found by its neighbors all of the time [32]. PSO has attractive characteristics. For example, the knowledge of good solutions is retained by all particles because of the use of an archive. Additionally, there is constructive cooperation among the particles, and particles in the swarm share information with each other [33]. The procedure of the GAPSOBP method is illustrated in Fig 4.

A hybrid GAPSO-based BPNN is expected to have a better prediction accuracy. This expectation is confirmed in a study [32] in which the authors demonstrated that GAPSOBP has a faster convergence speed and a higher prediction precision than GABP in terms of network traffic prediction. In this study, we investigate which optimized BPNN performs best in terms of event-related change detection. We test and verify the different methods based on two real-world studies in Section 6 to answer Question 2 in Section 2.

## 5. Experimental design

### 5.1 Experimental parameters

#### 5.1.1 Input selection and parameter preprocessing

As previously mentioned, the organizational network is represented as network measures. However, it is quite challenging to select useful measures to distinguish anomalous networks from normal networks. There are many network measures that can be calculated from a given network, including graph-level measures, e.g., density, and node-level measures, e.g., degree centrality. In this paper, for exposition purposes, we focus on graph-level measures rather than node-level measures to investigate the overall changes in the network as opposed to changes at the level of individual agents. Most statistical analyses using SNA focus on identifying the average responses in the measures [16]. Therefore, the node-level measures are transformed into graph-level measures by averaging the measures across the whole network. In previous studies [4,5,23], measures are manually selected subjectively, and the selection may not be due to the significant characteristics of the network. Additionally, social networks are occasionally built on incorrect or incomplete information; in practice, these networks appear to have a relatively lower accuracy and sensitivity. Therefore, there is no consensus regarding which single network measure to include in a model that will lead to a better performance. In this paper, we select a multi-dimensional measure vector and use the following criteria to select significant measures. We propose several criteria for measure selection. We select measures that 1) change significantly over time, 2) exhibit a strong correlation between the temporal trends of the selected measures and organizational behaviors, and 3) have been commonly used in previous studies. To improve accuracy, measures are normalized within [0, 1] using Eq (5). *X*_max_ and *X*_min_ represent the maximum and minimum value of each input measure.

$$X=\frac{X-X_{\min}}{X_{\max}-X_{\min}}$$ (5)

#### 5.1.2 Selection of hidden neuron

The selection of the hidden neuron is of great importance in the process of constructing the neural network architecture. In this paper, a single hidden layer is used. Theoretically, one hidden layer in a neural network with a sufficient number of hidden neurons is capable of approximating continuous functions [34]. The number of neurons in the hidden layer can be determined by Eq (6) [35]:

$$N_{hidden}=\left\lfloor \sqrt{N_{in}+N_{out}}+a\right\rfloor$$ (6)

where *N*_*hidden*_, *N*_*in*_, and *N*_*out*_ denote the number of nodes in the hidden layer, input layer, and output layer, respectively. A number in [1, 10] was suggested for parameter *a*.

### 5.2 Evaluation of performance

#### 5.2.1 Baselines

First, to demonstrate that our optimized BPNNs are better and that the combination of GA and PSO is logical, we set a simple BPNN as the baseline. As discussed in Section 4, simple BPNNs have drawbacks; however, no studies have been performed in the area of dynamic social network change detection. Therefore, we must determine whether the optimized BPNNs are better than a simple BPNN. Then, the results are compared using the following six state-of-the-art methods: Statistical Process Control (SPC), Logistic Regression (LR), Local Outlier Factor (LOF), Decision Trees (DT), Support Vector Machines (SVM), and One-Class SVM (OC-SVM). Of these methods, LR, DT and SVM are classification methods using supervised information, whereas LOF, OC-SVM, and SPC are all unsupervised anomaly detection methods.

#### 5.2.2 Evaluation strategies

We use *ROC* and *AUC* to evaluate the performance of our methods and the simple BPNN. *ROC* is a graphical plot that illustrates the performance of a binary classifier system. The curve is created by plotting the *TPR* (True Positive Rate) against the *FPR* (False Positive Rate) at various threshold settings. The *AUC* is the area under the *ROC*. An *AUC* between 0.5 and 0.7 represents low precision; an *AUC* between 0.7 and 0.9 indicates good precision; and an *AUC* between 0.9 and 1 suggests better precision.

According to *Youden’s* index (*J*), we can obtain the optimum cut-off point, and then, the neural networks could yield a dichotomous result. Then, we use *Precision*, *Recall*, and *F-score*, which are computed using the test set, to evaluate the performance of the different methods. The *Precision* is the number of correctly identified positives divided by the number of identified positive instances. The *Recall* is the number of correctly identified positives divided by the number of all positive instances in the test set. The *F-score* [36] is defined by Eq (7).

$$F\text{-}score=2\times \frac{precision\times recall}{precision+recall}$$ (7)

These evaluation strategies are selected to estimate the effectiveness of the classification approach presented in Section 3.3 and the optimized neural network models proposed in Section 4 and finally to answer Question 3, which was raised in Section 2.

## 6. Results and discussion

In this section, we present experiments using two datasets and discuss the results according to our proposed framework.

### 6.1 Enron e-mail network

#### 6.1.1 Data description

Within only a few months, the Enron Corporation, which used to be one of the world’s largest corporations, underwent a meltdown and bankruptcy, raising many serious and troubling issues. When Enron collapsed in 2001, approximately 500,000 internal e-mails became public. At that time, the timeline of Enron’s collapse and the significant events related to that collapse were reported by the Washington Post [37]. Shetty and Adibi [38] cleaned Enron’s data and loaded these data into a *MySQL* database (S1 Text). The time period of the dataset covers May 1999 to April 2002. All information of interest can be extracted from the database.

**Fig 5:**
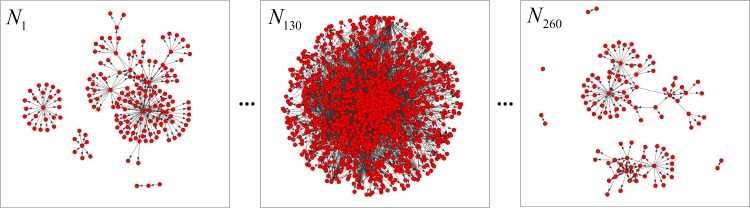
Examples of snapshots of the Enron networks.

We extracted the *senders*, *receivers*, and *date* information from the dataset and then applied the information to build longitudinal social networks based on the mechanism introduced in Section 3.2. The network analysis tool *ORA* [39] was used to build the networks based on the relationship between *senders* and *receivers*. The networks were weighted directed social networks with links from *senders* to *receivers*. We sampled 260 days between May 1999 and April 2002 and built a time sequence of 260 social networks, which were labeled with IDs of *N*_1_,*N*_2_,…,*N*_260_. Fig 5 provides examples of the network snapshots of the Enron networks.

#### 6.1.2 Experimental results

**Fig 6:**
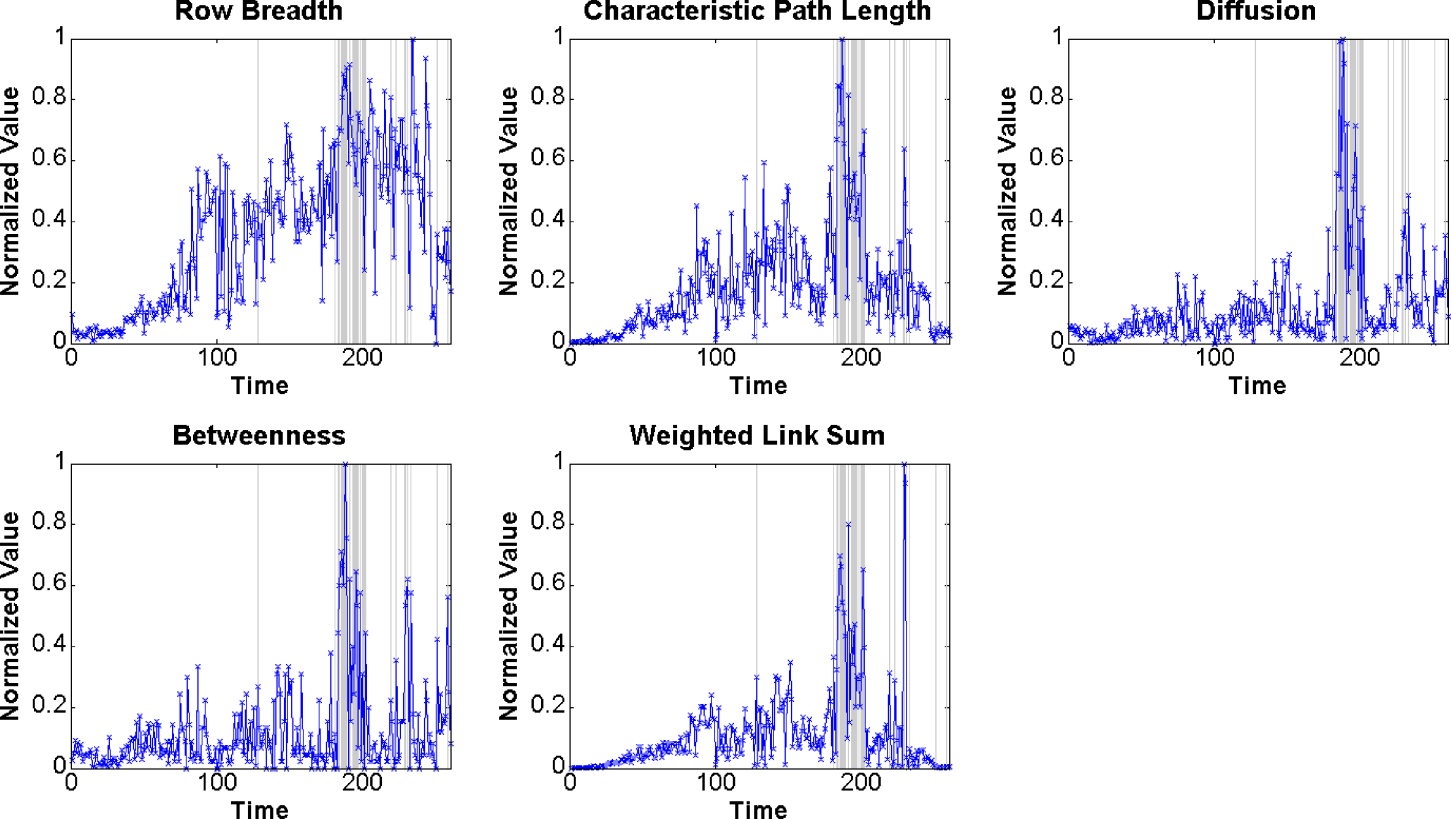
Trends of selected measures of the Enron networks.

Based on the selection criteria presented in Section 5.1.1, we selected *Row Breadth*, *Characteristic Path Length*, *Diffusion*, *Betweenness Network Centralization*, and *Weighted Link Sum* as the input variables. The selected measures are typical features of the Enron networks, and these measures could express the structure and interaction patterns of the organization to a certain extent. For example, *Row Breadth* is defined using the out degree to measure the frequency of e-mails sent by each node, and *Weighted Link Sum* is used to measure the intensity of the communication. Both are key factors of directed networks. Eq (6) is used to normalize the selected measures within [0, 1]. The trend of each selected measure is shown in Fig 6.

Employing Eq (1), dichotomous values of 0/1 were assigned for the time sequence based on whether there was an event at Enron Corporation. We referenced the *Timeline of Enron’s Collapse* in the Washington Post [37] for a list of external events. For example, on October 17, 2001, “*SEC sends a letter to Enron asking for information after the company reported hundreds of millions of dollars in third-quarter losses”*. This loss was a verified event for Enron Corporation. Then, the corresponding time point *T*_185_ was labeled “1”. Under this rule, the time sequence was converted into a 0/1 set in which 26 time points were labeled “1”, and 234 time points were labeled “0”. This set was imported into the output layer of the neural network. For the number of neurons in the hidden layer of Eq (6), *a* = 10 was applied because it performed well after a set of trial and error tests. Thus, as *N*_*in*_ = 5 and *N*_*out*_ = 1, *N*_*hidden*_ = 12, and the architecture of the neural network was 5-12-1.

**Fig 7:**
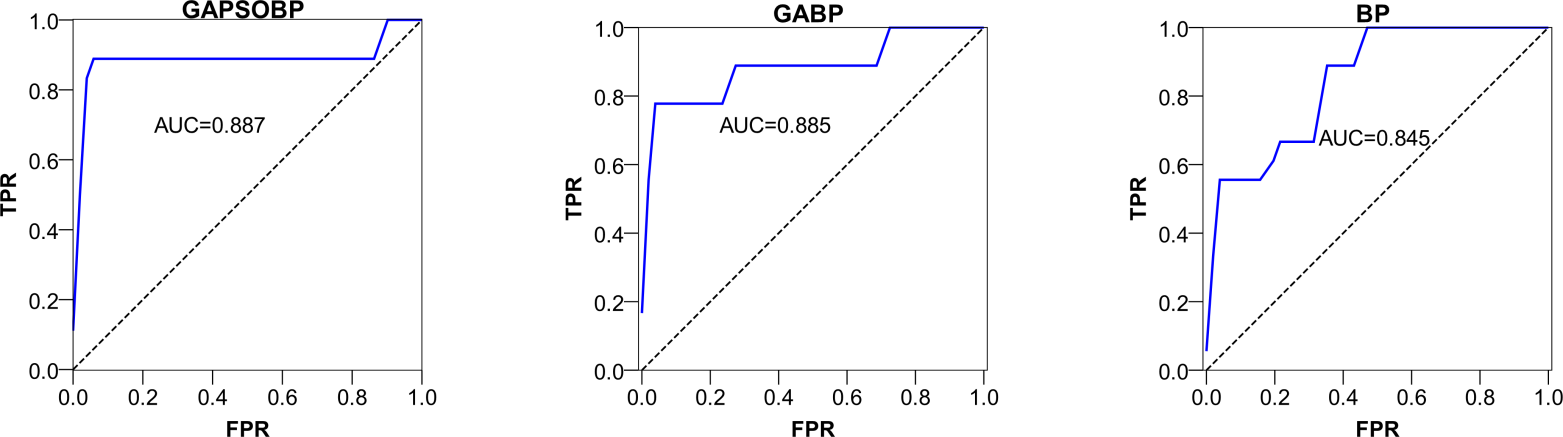
*ROC* and *AUC* using different neural network models of the Enron networks.

For all methods with parameters, we divided the time sequence networks into subsets for the model training and test. The data of the first 200 time points were used as the training samples, of which 17 were labeled “1”, and 183 were labeled “0”. The data of the later 60 time points were used as the test samples, of which 9 were labeled “1”, and 51 were labeled “0”. We then applied the GABP and GAPSOBP neural networks to detect the event-related changes in the Enron networks. After running GABP, GAPSOBP, and BP 100 times, the detection results were obtained. Fig 7 provides the *ROC* and *AUC* of the results of GAPSOBP, GABP, and BP. According to the plots, we found that the *AUC* of GAPSOBP was 0.887, which is greater than the corresponding values of GABP and BP. Using this criterion, GAPSOBP performed better than GABP and BP, whereas GABP performed better than BP. Because the values were all between 0.7 and 0.9, the supervised neural network models delivered good precision in change detection.

Using *Youden’s* index, the best cut-off values for GAPSOBP and GABP were then selected. Under these threshold values, we could obtain the detection results of the two optimized BPNNs. The result of GAPSOBP shows that changes had occurred at time points *T*_201_, *T*_202_, *T*_229_, and *T*_230_, i.e., on 2001/11/13, 2001/11/18, 2002/2/2, and 2002/2/4. GABP showed that changes had occurred at time points *T*_201_, *T*_228_, and *T*_229_, i.e., on 2001/11/13, 2002/1/31, and 2002/2/2.

Finally, we performed the SPC, LR, LOF, DT, SVM, and OC-SVM runs similarly. Table 1 summarizes the performance of our proposed BPNNs and the baseline methods. According to Table 1, we find that, although the *Precisions* of some baseline methods were as high as those of our methods, the optimized BPNNs significantly improved the *Recall* and *F-score*, and GAPSOBP had the best performance.

**Table 1:** Performance comparison of the Enron networks.

Method	*Precision*	*Recall*	*F-score*
**GAPSOBP**	0.571	0.444	0.499
**GABP**	0.600	0.333	0.428
**BP**	0.500	0.333	0.400
**SVM**	0.364	0.444	0.400
**DT**	0.333	0.111	0.167
**OC-SVM**	0.107	0.333	0.162
**SPC**	0.667	0.222	0.333
**LR**	0.429	0.333	0.375
**LOF**	0.500	0.222	0.307

To further verify our methods, we conducted a study using Al-Qaeda networks that aimed to implement the optimized neural networks to another dataset to evaluate the validity and generality of our methods.

### 6.2 Al-Qaeda network

#### 6.2.1 Data description

**Fig 8:**
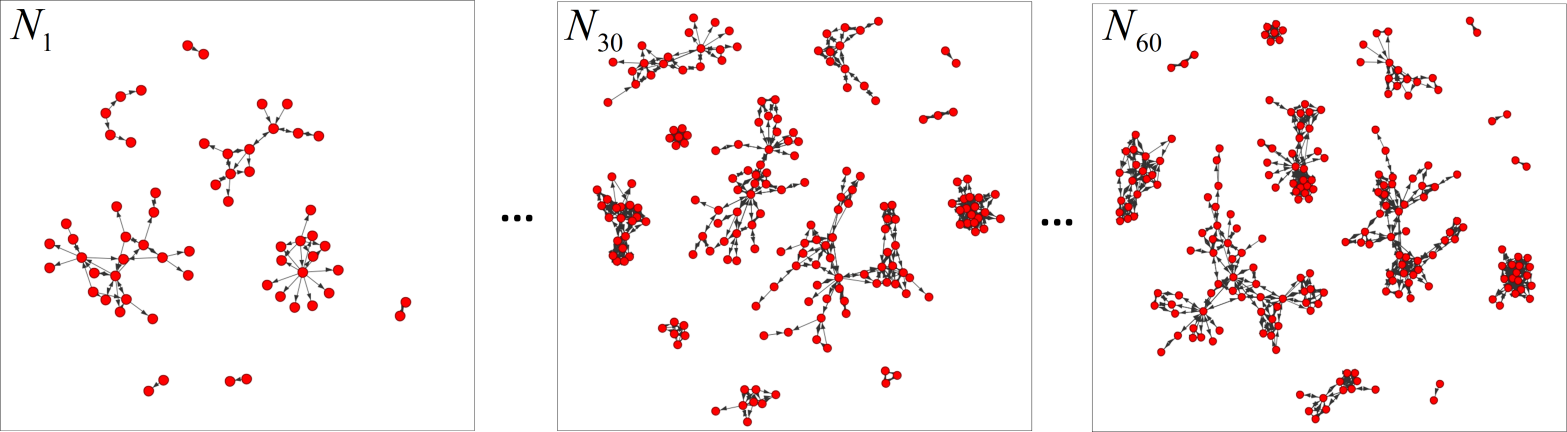
Examples of snapshots of the Al-Qaeda networks.

Al-Qaeda is a global militant Islamist organization founded by Bin Laden that has been designated a terrorist organization by the United Nations Security Council. Al-Qaeda has executed many attacks on civilian and military targets in various countries, including the 1998 US embassy bombings and the September 11 attack. The data used in this study are retrieved from the *John Jay & ARTIS Transnational Terrorism Database (JJATT)* [40] (S1 Text). The network data in the *JJATT* describe the relationships that individuals shared in select attack networks. The data include the network members and the operational ties among the members. The data also pool the relationships of the individuals associated with the terrorist attacks (events). For the Al-Qaeda dataset, we initially applied the information extracted from the dataset to build longitudinal social networks. We built a sequence of networks quarterly (every three months) from 1989 to 2003. Consequently, 60 networks with IDs of *N*_1_,*N*_2_,…,*N*_60_ were established as shown in Fig 8. In each network, the nodes are terrorists, and the links are the relationships among the terrorists. The operational ties are willful choices made by the terrorists for political and strategic reasons [41]. During the process of the operation, the terrorists connect and reconnect dynamically with each other in pursuit of operational success. For example, foot soldiers take orders from a higher authority to recruit new members or obtain financial support. Therefore, the Al-Qaeda networks are also weighted directed social networks.

#### 6.2.2 Experimental results

**Fig 9:**
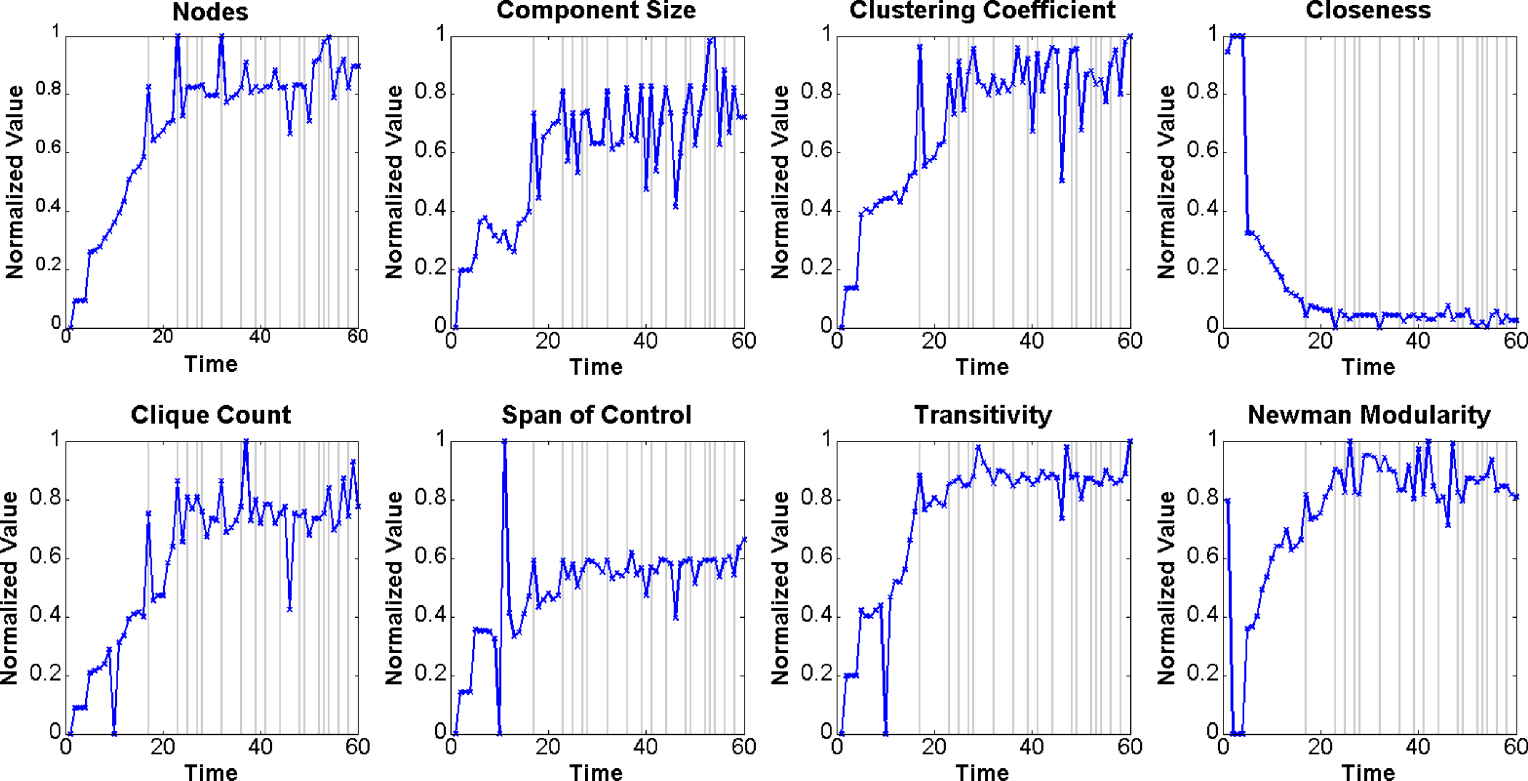
Trends of selected measures of the Al-Qaeda networks.

In this study, we considered the terrorist attack incidents external events of the Al-Qaeda organization. Similarly, we selected *Nodes*, *Component Size*, *Clustering coefficient*, *Closeness*, *Clique Count*, *Span of Control*, *Transitivity*, and *Newman Modularity* as the input variables. The selected measures are also typical features of the Al-Qaeda networks. For example, *Span of Control*, which is defined as the average number of out links per node with non-zero out degrees [39], is used to measure the numbers of orders from the authorities. Increasing sizes of the organization, denoted by *Nodes* and *Component Size*, tend to accelerate the production and frequency of violent events [42]. The trends of the normalized measures are shown in Fig 9.

Using the same method as that used in the Enron study, we referenced a list of terrorist attacks by Al-Qaeda to assign dichotomous values for the time sequence. Overall, 17 time points were labeled “1”, and 43 time points were labeled “0”, indicating that there were 17 time points during which the organization took action for attacks. The 0/1 set was imported into the output layer of the neural network. Using Eq (7), *a* = 9 was applied, and *N*_*hidden*_ = 12 was obtained. Therefore, the architecture of the neural network was 8-12-1. Data of the first 40 time points were used as training samples, of which 8 were labeled “1” and 32 were labeled “0”. The data of the later 20 time points were used as the test samples, of which 9 were labeled “1”, and 11 were labeled “0”.

**Fig 10:**
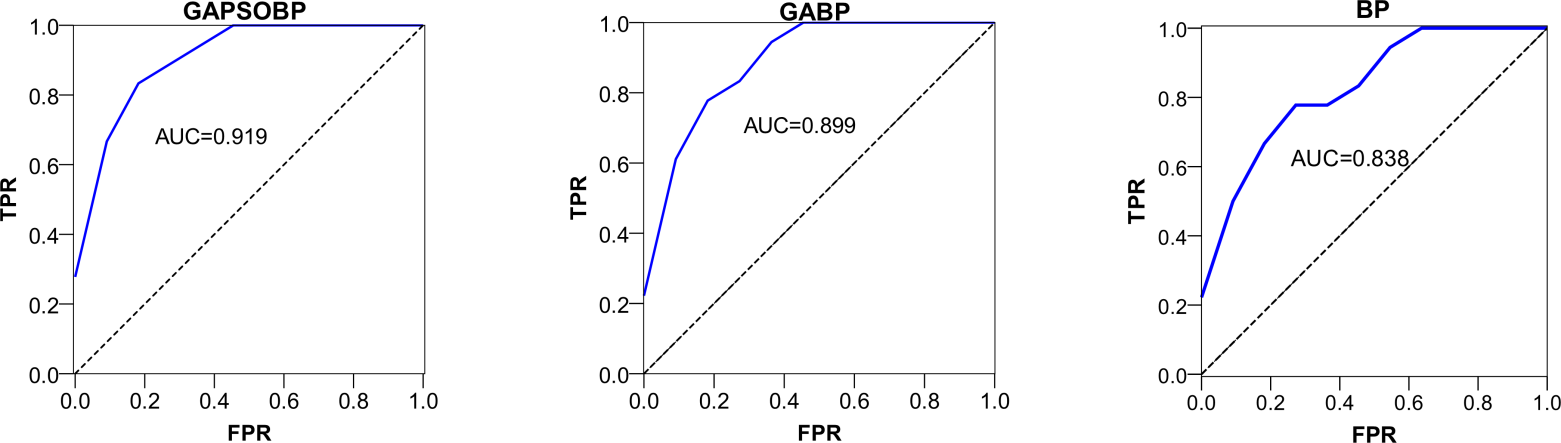
*ROC* and *AUC* using three different neural networks for the al-Qaeda networks.

After running GABP and GAPSOBP 100 times, the detection results were obtained. Fig 10 illustrates the *ROC* and *AUC* using our methods with a simple BP. Similarly to the Enron study, we found that GAPSOBP had the best performance, whereas GABP performed better than BP. Because the values were all between 0.7 and 0.9, we show that the supervised neural network models provided good precision.

Using *Youden’s* index, we found that event-related changes had occurred at time points *T*_41_, *T*_44_, *T*_45_, *T*_48_, *T*_49_, *T*_51_, *T*_53_, *T*_54_, *T*_58_, and *T*_59_. All baseline methods are run using the same data. Table 2 compares the performance of the different methods. According to the table, we find that the *Precision*, *Recall*, and *F-score* using BPNN were exceptional, particularly when optimized using GA and PSO. Obviously, our methods performed better than the baselines, and GAPSOBP had the best performance.

**Table 2:** Performance comparison of the Al-Qaeda networks.

Method	*Precision*	*Recall*	*F-score*
**GAPSOBP**	0.875	0.778	0.824
**GABP**	0.727	0.889	0.799
**BP**	0.700	0.778	0.737
**SVM**	0.625	0.556	0.588
**DT**	0.333	0.333	0.333
**OC-SVM**	0.286	0.444	0.348
**SPC**	0.714	0.556	0.625
**LR**	0.400	0.444	0.401
**LOF**	0.600	0.333	0.428

### 6.3 Discussion

By testing our proposed methods against baseline methods for detecting event-related changes in two studies, we obtained several interesting observations that confirm our research motivation and answer our research questions.

#### (1) Networks with event-related changes have structure patterns that are different than those of normal networks

This observation verifies the basics of this study, i.e., a correlation exists between the social networks and events. Therefore, the definition of event-related change in organizational networks is reasonable, and the detection of such changes is possible. Fig 6 and Fig 9 highlight the gap between the measures with and without event-related changes. To explain this phenomenon, the selected measures were divided into the following three categories according to their meanings: scale factor, clustering factor, and distance factor. The scale factor includes *Node Count*, *Span of Control*, *Weighted Link Sum*, and *Row Breadth*. According to the change in the scale factor, we found that when an event-related change in the network occurred, the number of members (nodes) in the organization grew. Consequently, the total number of connections (links) among the members increased. The clustering factor includes *Component Size*, *Clustering coefficient*, *Clique Count*, *Transitivity*, and *Newman Modularity*. We found that when event-related changes occurred in a network, more subgroups appeared, and within each subgroup, both the nodes and links increased. The distance factor includes *Closeness*, *Betweenness*, *Diffusion*, and *Characteristic Path Length*. By examining the changes in the distance factors, we found that the average distance between every two nodes grew as the closeness decreased. Overall, there was a specific structure pattern in the organizational network when external events occurred, and our methods had the ability to recognize that pattern.

To verify that the given external events are the reasons for the given changes in the measures, we further apply the Student’s t-test with 95% confidence (*p-value* <0.05). Table 3 shows the *p-value*s of the t-tests performed to compare the measures between the normal and event-related change networks. We found that the measure differences between the two classes are statistically significant. This finding provides evidence regarding the rationality of the selected measures.

**Table 3:** Comparison of selected measures between event-related change networks and normal networks.

Dataset	Network measures	Event-related change		Normal		*p-value*
		Mean	SD	Mean	SD	
**Enron**	*Row Breadth*	0.129	0.033	0.066	0.045	1.2E-10
	*Characteristic Path Length*	14.312	6.646	4.367	3.437	4.9E-08
	*Diffusion*	0.044	0.025	0.008	0.007	1.4E-07
	*Betweenness*	0.021	0.010	0.004	0.004	5.3E-09
	*Weighted Link Sum*	10138.115	6197.379	2103.696	2307.294	6.2E-07
**Al-Qaeda**	*Nodes*	204.765	13.661	156.767	46.001	0.0002
	*Component Size*	17.707	1.259	12.443	2.881	8.7E-09
	*Clustering coefficient*	0.365	0.021	0.254	0.096	4.6E-05
	*Closeness*	0.193	0.015	0.305	0.195	0.0357
	*Clique Count*	97.529	5.604	65.698	32.042	0.0002
	*Span of Control*	4.664	0.088	4.025	0.868	0.0068
	*Transitivity*	0.518	0.008	0.410	0.152	0.0089
	*Newman Modularity*	0.846	0.005	0.830	0.038	0.0455

#### (2) GAPSOBP neural network is the most valuable for event-related change detection in an organizational network

By examining the *Precision*, *Recall*, and *F-score* shown in Tables 1 and 2, GAPSOBP provided very good results for more known external events than the other methods. By examining the results more thoroughly, we found that GAPSOBP performed better than GABP in each of the two cases. As shown in both Fig 7 and Fig 10, we found that the *AUC* of the optimized BPNNs (GABP and GAPSOBP) had a greater value than that of the simple BPNN, but the difference was not significant. By further comparing the *F-score*, GAPSOBP performed significantly better than BPNN, whereas the gap between GABP and BPNN remained insignificant. This observation illustrates that the combination of the hybrid heuristic methods can effectively reduce the false-positives rate. The precisions of all anomaly detection methods are very low. SPC-based change detection methods only using a single network measure performed poorly, yielding high false-negative rates. This poor performance is likely the result of a single network measure discarding much of the specific information that can be crucial for change detection. Moreover, SPC, LOF, and OC-SVM were unsupervised methods; these methods could only identify large structural changes. BPNN performed better than LR largely because of the latter’s single-layer perception. In general, the supervised classification methods performed better than the unsupervised methods.

The Al-Qaeda study suggests that our optimized models are able to detect change even when the information is incomplete. The Al-Qaeda data is based on open source information [40], and inevitably, it is an incomplete representation of the interactions in that terrorist network. The limitations of the terrorist data make it difficult to determine the changes in the organizational behavior because we cannot be sure that we included all communication and cooperation networks. However, even using this dataset, we are able to systematically detect key changes, which highlights the value of the proposed models.

#### (3) Optimized BPNN has sufficient feasibility and generality for event-related change detection

Our methods had a high level of effectiveness. In both studies, the numbers of selected network measures were 5 and 8, respectively. These multidimensional measures were selected as the input variables and can represent the whole networks adequately. As discussed in Section 4, the optimized BPNN achieved a high accuracy with the ability to address multiple inputs and overcame the local convergence problem for nonlinear optimization problems. Meanwhile, the GAPSOBP and GABP neural networks were applied to two datasets and yielded good results. We found that the optimized BPNN model has a high generality. No need exists to establish a specific model for the research object. The models have universality toward different types of network topologies regardless of whether the networks are sparse or dense. Even when studying Al-Qaeda, in which the networks had several components and a decentralized structure [43], our methods still functioned well. Regardless of the type of network (i.e., an e-mail network or a social network between persons), good results were obtained using our methods.

## 7. Conclusions and future directions

The organizational internal structure is correlated with the external behavior change to a certain degree. By detecting the changes in the dynamic organizational networks, we can rapidly identify the underlying events of the organization. In this study, we defined event-related change in organizational networks and proposed a framework for detecting such changes. We applied neural network models that were optimized using hybrid heuristic methods as classification models. Two real-world studies were presented in this paper and demonstrated that the proposed methods could allow analysts and researchers to detect event-related changes in dynamic network data, which forcefully answered our research questions.

Compared with the baseline methods, our methods appear to have an obvious advantage because of their higher effect and precision. The optimized BPNN models could address more than one network measure without requiring the specific structure or distribution degree of the network. Their strength is also evident because they are well trained after numerous runs to recognize the pattern of networks regardless of low-quality data or relatively stable structural patterns. Most importantly, our methods can effectively utilize the supervised information by considering the correlation. In practice, our methods can be particularly useful for early warning and faster responses to both positive and negative organizational behaviors. Our methods can at least help recognize the dynamic pattern of the social organization.

In summary, we make the following contributions in this paper. (1) We introduce the notion of event-related change in organizational networks. Such a definition tightly integrates organizational behavior and networks with respect to both the temporal and structure dimensions. (2) We design an effective classification based event-related change detection framework that combines SNA, anomaly detection, and machine learning techniques (BPNN). The framework is based on the use of certain critical events to facilitate our ability to compute and reason about novel behavior-oriented measures, which can offer new and interesting insights for the characterization of dynamic behavior in these interaction networks. (3) We propose optimized BPNNs using heuristic methods according to the features of the study content. In addition, the social network-based change detection could be a potential application domain for artificial neural networks.

Social network analysis and pattern recognition have become increasingly popular and practical in recent years. Future work should expand our technique to a decision support system for a practical application. For instance, such a system could help security personnel obtain an early warning signal for terrorist events and respond faster to potential terrorist activities. Future research should also focus on improving the neural network based on the characteristics of the event-related change detection problem. Understanding the different types of changes in organization behavior and developing better integration mechanisms are urgently needed. In the future, we plan to further our studies on prediction (or early detection) of the external event. As previous studies [4,36,44] have highlighted, the current behaviors of an organization can be captured by certain attributes of the organization in the past history. It is possible to predict the future events of an organization. Therefore, in addition to current detection, early detection should also be implemented to provide an early awareness of a critical, yet subtle change in the organization behavior.

## Supporting information

S1 Text URLs of the datasets. (TXT)
